# Cooperative Safe Trajectory Planning for Quadrotor Swarms

**DOI:** 10.3390/s24020707

**Published:** 2024-01-22

**Authors:** Yahui Zhang, Peng Yi, Yiguang Hong

**Affiliations:** 1 Department of Control Science and Engineering, Tongji University, Shanghai 201804, China; zhangyahui680@tongji.edu.cn (Y.Z.); yghong@iss.ac.cn (Y.H.); 2 Shanghai Research Institute for Intelligent Autonomous Systems, Shanghai 201210, China

**Keywords:** trajectory planning, model predictive control, alternating direction multiplier method, differential flatness, control barrier function, quadrotor swarms

## Abstract

In this paper, we propose a novel distributed algorithm based on model predictive control and alternating direction multiplier method (DMPC-ADMM) for cooperative trajectory planning of quadrotor swarms. First, a receding horizon trajectory planning optimization problem is constructed, in which the differential flatness property is used to deal with the nonlinear dynamics of quadrotors while we design a relaxed form of the discrete-time control barrier function (DCBF) constraint to balance feasibility and safety. Then, we decompose the original trajectory planning problem by ADMM and solve it in a fully distributed manner with peer-to-peer communication, which induces the quadrotors within the communication range to reach a consensus on their future trajectories to enhance safety. In addition, an event-triggered mechanism is designed to reduce the communication overhead. The simulation results verify that the trajectories generated by our method are real-time, safe, and smooth. A comprehensive comparison with the centralized strategy and several other distributed strategies in terms of real-time, safety, and feasibility verifies that our method is more suitable for the trajectory planning of large-scale quadrotor swarms.

## 1. Introduction

In recent years, with the rapid development of communication, computing, and automation technologies, intelligent UAV swarm systems inspired by the behavior of biological swarms have received extensive attention from researchers and practitioners [1]. An intelligent UAV swarm system is a holistic system composed of a group of UAVs capable of accomplishing complex tasks through cooperation and information sharing [2]. UAV swarm systems have advantages in terms of timeliness, economy, and functionality [3]. Consequently, they have been widely used in both civil and military fields [4], such as infrastructure inspection [5], logistics transportation [6], and search and rescue [7]. Among them, trajectory planning, as a crucial and challenging component for UAV swarms to perform tasks, needs to ensure the safety of a smooth trajectory from the initial position to the target position under the dynamics constraints, especially in a confined space. Furthermore, coordination among the UAVs is crucial.

There is increasing research that has emerged to address this safety-critical challenging problem, including potential fields [8], velocity obstacles [9,10], dynamic windows [11], and inter-robot prioritization [12]. However, these classical methods do not pay much attention to the interaction among robots, making them ineffective for more complex environments. Recently, learning-based methods [13,14] have been proposed to consider inter-robot interaction to solve trajectory planning. However, they usually require high-quality training data for generalization and lose the interpretability of the internal decision-making process. The above problems can be overcome by constructing an optimization problem using the MPC framework to handle complex objective functions and constraints explicitly, as well as providing sequences of future state and control input information predictively [15,16,17].

**Figure 1:**
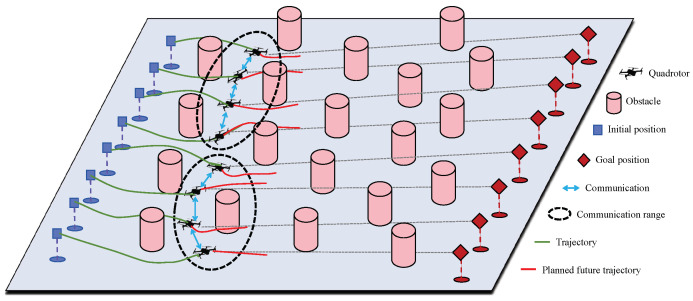
Illustration of the trajectory planning based on DMPC-ADMM for a quadrotor swarm in a crowded environment.

The major challenge of implementing MPC for the trajectory planning of quadrotor swarms lies in the difficulties of guaranteeing the real-time feasibility and safety of the system under various practical constraints. In this paper, we present a novel distributed trajectory planning algorithm for quadrotor swarms based on DMPC-ADMM, referring to Figure 1. Our contributions can be summarized as follows:

- A trajectory planning optimization problem for quadrotor swarms is constructed based on MPC, which uses the differential flatness property to handle the nonlinear dynamics of quadrotors. The dimension of the planning space is reduced compared to directly utilizing the nonlinear model. Additionally, we design a relaxed form of DCBF constraint to balance feasibility and safety. Due to the non-convexity of the DCBF, we linearize the DCBF at each time step and use an iterative convex optimization scheme to improve the solution’s efficiency.
- The high-dimensional optimization problem is decomposed to construct a fully distributed trajectory planning algorithm based on ADMM. In this distributed algorithm, the quadrotors within the communication range reach a consensus on future trajectories through cooperation, thus enhancing the safety of trajectories. To further improve communication efficiency, we design an event-triggered mechanism to reduce the communication overhead.
- The simulation results verify that our method can generate safe and smooth trajectories online under limited communication range, collision avoidance, and dynamic constraints. The comparison with the centralized strategy and several other distributed strategies confirms that our method is more suitable for large-scale quadrotor swarms.

The remainder of this paper is organized as follows. In Section 2, we mainly review the literature on the trajectory planning of UAVs. In Section 3, we introduce the differential flatness and DCBF. The quadrotor swarm trajectory planning problem based on MPC and DCBF is formulated in Section 4. In Section 5, we provide the distributed algorithm to solve the optimization problem via ADMM and design an event-triggered mechanism to reduce the communication overhead. We present the simulation experiments and results in Section 6, followed by concluding remarks in Section 7.

## 2. Related Work

Ensuring the safety of generated trajectories has consistently been a central issue in the field of multi-robotics, as well as a prerequisite for successful execution of tasks. As noted in [18], the existing literature often adopts Euclidean norms to model distance constraints, which are activated only when the reachable set intersects with the obstacles, resulting in the robot taking action only when it is close to them. To overcome this shortcoming, Ref. [18] combined CBF with MPC to avoid obstacles at an early stage and enhance the safety of the trajectory. The MPC-CBF formulation was also investigated on different platforms, including unmanned aerial vehicles [19,20] and autonomous vehicles [21].

The object of interest in this paper is a group of quadrotors whose underactuation and intrinsic instability make generating safe trajectories challenging. An effective solution involves leveraging the differential flatness property of quadrotors, as introduced in [22], to simplify the optimization problem while preserving the nonlinear dynamics of the quadrotors. This approach was successfully applied to UAV trajectory planning in crowded environments [23], as well as avoiding obstacles [24]. Specifically, Ref. [19] used CBF constraints to ensure trajectory safety for multi-quadrotor systems based on differential flatness. In this paper, we propose a distributed algorithm for the trajectory planning of quadrotor swarms with the MPC-CBF formulation.

**Figure 2:**
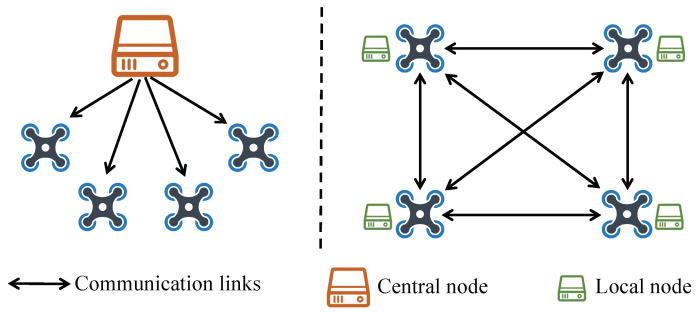
Centralized design (**left**) vs. our distributed design (**right**) for cooperative trajectory planning of quadrotor swarms.

UAV swarm trajectory planning is a complex optimization problem involving multiple constraints, variables, and nonlinear effects. Several existing studies on the trajectory planning of UAVs are summarized in Table 1. Centralized methods [24,25,26] have been proposed for the trajectory planning of UAV swarms. Kumar et al. [24] resolved the heterogeneous UAV formation reconfiguration and trajectory planning based on mixed integer quadratic programming (MIQP). Ref. [25] considered the collision-free trajectory generation for multiple UAVs in 3D space as a nonconvex optimization problem, which can be solved by sequential convex programming (SCP). These centralized approaches require the presence of a central node capable of acquiring the state information of the entire system and transmitting the planning results to each UAV. Nevertheless, one of its disadvantages is the large amount of computation, and once the center node fails, the whole system stops working, resulting in poor real-time and scalability. Therefore, the distributed methods have attracted much research attention in recent years. Compared to the centralized ones, each UAV in the distributed framework uses peer-to-peer communication to compute its trajectory without the need for a central node, referring to Figure 2. Hence, the distributed framework is scalable and robust in the face of unexpected node and link failures.

Distributed Model Predictive Control (DMPC) [27] has found successful applications in various networked systems ranging from electric power networks [28] to multi-robot systems [29,30,31]. It is an appealing option for distributed trajectory planning. Borrelli et al. [29] addressed the UAV formation problem based on a decentralized linear MPC approach that guaranteed collision avoidance under constraints. Luis et al. [31] developed a DMPC algorithm for online point-to-point flight trajectory generation in multi-UAV scenarios, which employed on-demand collision avoidance and event-triggered replanning. However, the second-order dynamics did not take into account the characteristics of quadrotors, and the safe distance constraint was approximated by a first-order Taylor expansion of the Euclidean distance. These limitations made it challenging to ensure trajectory safety in confined environments. To solve the quadrotor navigation problem, an online decentralized obstacle avoidance algorithm based on differential flatness and MPC was proposed in [30], where Optimal Reciprocal Collision Avoidance (ORCA) was employed for obstacle avoidance. However, the quadrotors lack cooperation to reach a consensus on their future trajectories, potentially leading to collisions in dense environments.

**Table 1:** Existing studies on the trajectory planning of UAVs.

References	UAV Model	Methods	Collision Avoidance	Evaluation
Kumar et al. [24]	Differential flatness	MIQP and downwash effect	Yes	**Centralized methods:** optimal global planning, poor real-time, high computational complexity, poor scalability
Augugliaro et al. [25]	Differential flatness	Discrete planning and continuous refinement	Yes	
Preiss et al. [26]	Second-order dynamics	SCP and posteriori vehicle-specific feasibility check	Yes	
Borrelli et al. [29]	Second-order dynamics	MILP and inter-vehicle coordination rules	Yes	**Distributed methods:** good real-time, strong robustness, suitable for large-scale swarms
Arul et al. [30]	Linear flat model	ORCA, downwash effect, and flatness-based feedforward linearization	Yes	
Luis et al. [31]	Second-order dynamics	On-demand collision avoidance	Yes	

Distributed optimization theory and its applications have gained increasing attention in recent years. The alternating direction multiplier method (ADMM) [32], as one of them, has been proven to show significant advantages in terms of convergence speed and computational efficiency in multi-robot task scenarios [33]. A series of examples combining MPC and ADMM approaches for multiple robots [3,34,35,36] illustrate the effectiveness and scalability of such a distributed framework. For instance, Ref. [34] proposed a distributed MPC method utilizing ADMM decomposition to coordinate the control problem of waterborne AGVs. Chen et al. [35] employed MPC and ADMM to achieve formation navigation of multiple vessels under environmental perturbations. In [3], a flocking control framework based on MPC and ADMM was presented to enable multi-vehicle systems to track desired trajectories while considering limited communication distances, collision avoidance, and bounded speed and control inputs. Compared to the methods above, we address a more complex quadrotor scenario that takes into account the quadrotor’s dynamics. Moreover, we utilize the linearized DCBF instead of the Euclidean norms and incorporate MPC to ensure the real-time generation of safe trajectories.

## 3. Preliminaries

In this section, we present an overview of differential flatness and DCBF. We highlight the linear flat model of the quadrotor and a relaxed form of the DCBF constraint. In addition, the main symbols used in this paper are defined in Abbreviations.

### 3.1. Differential Flatness and Quadrotor Dynamics

A nonlinear system $\dot{x}=f\left(x,u\right)$ is considered differentially flat if it can be described using a set of differentially independent variables $\zeta \in R^{m}$ called flat output, i.e., the state and input of the system can be expressed as algebraic functions of the flat output and its finite-order derivatives [37]. The definition is as follows:

(1) $$\zeta =\Phi (x,u,\dot{u},\ldots ,u^{\left(p\right)}),$$

(2) $$x=\Psi_{x}\left(\zeta ,\dot{\zeta },\ldots ,\zeta^{\left(q-1\right)}\right),$$

(3) $$u=\Psi_{u}\left(\zeta ,\dot{\zeta },\ldots ,\zeta^{\left(q\right)}\right),$$

where Φ , $\Psi_{x}$ , and $\Psi_{u}$ are smooth functions. Both $\Psi_{x}$ and $\Psi_{u}$ are also called endogenous transformations of the system. Here, *p* and *q* are the maximum orders of the derivatives of u and ζ required to describe the system. The quadrotor has been shown to have the differential flatness property [22] with a flat output $\zeta ={\left[x,y,z,\psi \right]}^{T}$ .

Consider a quadrotor with its control input $u=(T,\phi_{cmd},\theta_{cmd},{\dot{\psi }}_{cmd})$ , where *T* is the commanded thrust, $\phi_{cmd}$ and $\theta_{cmd}$ are the commanded roll and pitch angles, and ${\dot{\psi }}_{cmd}$ is the commanded yaw rate. The state $x=(x,y,z,\dot{x},\dot{y},\dot{z},\phi ,\theta ,\psi )$ includes position, velocity, and roll, pitch, and yaw angles. As in [38], the inner-loop attitude dynamics are written as follows:

(4) $$\begin{array}{c} \dot{\phi }=\frac{1}{\tau_{\phi }}\left(k_{\phi }\phi_{cmd}-\phi \right),\\ \dot{\theta }=\frac{1}{\tau_{\theta }}\left(k_{\theta }\theta_{cmd}-\theta \right),\\ \dot{\psi }={\dot{\psi }}_{cmd}, \end{array}$$

where $k_{\phi },k_{\theta }$ and $\tau_{\phi },\tau_{\theta }$ are the gains and time constants of the roll and pitch angles, respectively. Then the following equation gives the relationship between T,ϕ,θ and flat output ζ .

(5) $$\begin{array}{cc} T & =m\frac{\ddot{z}+g}{\cos\phi \cos\theta },\\ \phi & =\arctan\left(\frac{\ddot{x}\sin\psi -\ddot{y}\cos\psi }{\ddot{z}+g}\cos\theta \right),\\ \theta & =\arctan\left(\frac{\ddot{x}\cos\psi +\ddot{y}\sin\psi }{\ddot{z}+g}\right). \end{array}$$

From (4) and (5), we can then compute u in terms of flat output ζ and its derivatives. Hence, we simplify the quadrotor trajectory generation process by seeking a sufficiently smooth flat output trajectory within a reduced planning space dimension.

Hagenmeyer et al. [37] introduced the notion of exact feedforward linearization based on differential flatness. The differential flat system $\dot{x}=f\left(x,u\right)$ can be transformed into an equivalent linear discrete-time flat model that can provide the benefit of reducing computation overload. The linear flat model of the quadrotor [38] is shown as:

(6) $$\begin{array}{cc} \; & z^{k+1}=Az^{k}+Bv^{k},\\ \; & \zeta^{k}=Cz^{k}, \end{array}$$

where

$$\begin{array}{cc} \; & A=\left[\begin{array}{cccc} I_{3\times 3} & I_{3\times 3}T_{s} & I_{3\times 3}\frac{T_{s}^{2}}{2} & 0_{3\times 1}\\ 0_{3\times 3} & I_{3\times 3} & I_{3\times 3}T_{s} & 0_{3\times 1}\\ 0_{3\times 3} & 0_{3\times 3} & I_{3\times 3} & 0_{3\times 1}\\ 0_{1\times 3} & 0_{1\times 3} & 0_{1\times 3} & 1 \end{array}\right],\;B=\left[\begin{array}{cc} I_{3\times 3}\frac{T_{s}^{3}}{6} & 0_{3\times 1}\\ I_{3\times 3}\frac{T_{s}^{2}}{2} & 0_{3\times 1}\\ I_{3\times 3} & 0_{3\times 1}\\ 0_{1\times 3} & T_{s} \end{array}\right],\\ \; & C=\left[\begin{array}{cccc} I_{3\times 3} & 0_{3\times 3} & 0_{3\times 3} & 0_{3\times 1}\\ 0_{1\times 3} & 0_{1\times 3} & 0_{1\times 3} & 1 \end{array}\right], \end{array}$$

and $z={\left[x,y,z,\dot{x},\dot{y},\dot{z},\ddot{x},\ddot{y},\ddot{z},\psi \right]}^{T}$ denotes the flat state of the quadrotor, including position, velocity, acceleration, and yaw angle. $v={\left[\dddot{x},\dddot{y},\dddot{z},\dot{\psi }\right]}^{T}$ denotes the flat input, including the third-order derivatives of the position and the yaw rate. In this paper, we consider (6) as the quadrotor dynamics model to generate a smooth collision-free trajectory.

### 3.2. Discrete-Time CBF

Consider a discrete-time dynamical system as

(7) $$x^{k+1}=f\left(x^{k},u^{k}\right),$$

where $x^{k}\in X\subset R^{n}$ denotes the state of the system at time *k*, $u^{k}\in U\subset R^{m}$ is the control input, and *f* is a continuous dynamics function. Obstacle avoidance requires the invariance of a trajectory with respect to a safe, connected set. Specifically, if the system (7) is safe with respect to a set C , then any trajectory starting inside the set C will remain inside it. The set C is defined to be a superlevel set of the continuously differentiable functions h:X→R .

(8) $$C=\{x\in X\subset R^{n}:h\left(x\right)\geq 0\}.$$

Here, C is called a safety set. The function *h* is a discrete-time CBF (DCBF) [18] if the following condition (9) is satisfied.

(9) $$\exists \;u^{k}\;s.t.\;\Delta h\left(x^{k},u^{k}\right)\geq -\gamma h\left(x^{k}\right),\;0<\gamma \leq 1,$$

where $\Delta h\left(x^{k},u^{k}\right):=h\left(x^{k+1}\right)-h\left(x^{k}\right)$ , and γ is a hyperparameter. The constraint (9) implies $h\left(x^{k+1}\right)\geq \left(1-\gamma \right)h\left(x^{k}\right)$ , i.e., the lower bound of h(x) decays exponentially at time *k* with the rate 1−γ . Incorporating a valid DCBF constraint (9) into an optimization problem ensures the safety of the generated trajectories. If γ decreases, the system becomes more capable of avoiding obstacles, but it might lead to an unfeasible problem. On the other hand, if γ increases, the problem is more likely to be feasible, but the trajectory rapidly approaches the boundary of the safe set ∂C , making the system unsafe. Therefore, the fixed γ is challenging to adapt to complex and changing environments; see [18] for details. To better balance feasibility and safety, we design a relaxed form of the DCBF constraint as follows:

(10) $$h\left(x^{k+1}\right)-\left(1-\gamma \right)h\left(x^{k}\right)+\omega^{k}\geq 0.$$

Here, the slack variable $\omega^{k}\in R$ will be adaptively optimized along with other variables in the optimization problem. The collision-free trajectory needs to consider both the neighbors and the obstacles in the external environment, so two types of safety constraints are added. One is the $DCBC_{ij}$ constraint between quadrotor *i* and quadrotor *j*, and the other is the $DCBC_{io}$ constraint between quadrotor *i* and obstacle o∈O . The formulation of $DCBC_{ij}$ and $DCBC_{io}$ are shown in the next section.

## 4. Problem Formulation

In this section, we present an undirected adjacency graph representing communication among quadrotors and then formulate a trajectory planning optimization problem based on MPC and DCBF.

### 4.1. Proximity Network

To realize cooperative trajectory planning, quadrotors need to communicate and exchange information with each other. In this paper, we use an undirected proximity graph $G^{k}=\left(V,E^{k}\right)$ to represent the communication topology of quadrotors at time *k*, where V:={1,2,…,N} is the set of vertices and $E^{k}\in V\times V$ is the set of edges. In graph $G^{k}$ , vertex *i* stands for quadrotor *i*. The edge $E_{ij}^{k}$ stands for a communication link between quadrotor *i* and quadrotor *j* at time *k*, as

(11) $$E^{k}=\left\{\left(i,j\right)|d_{ij}^{k}<\Delta^{\mathrm{dect}},\;i,j\in V,\;i\neq j\right\},$$

where $d_{ij}^{k}=∥p_{i}-p_{j}∥$ and $\Delta^{\mathrm{dect}}>0$ is the maximum distance that quadrotors can communicate. At time *k*, the neighbors of quadrotor *i* are defined as $N_{i}^{k}≜\left\{j|\left(i,j\right)\in E^{k}\right\}$ . The graph $G^{k}$ is time-varying due to the movement of the quadrotors. Therefore, $N_{i}^{k}$ may be ∅, i.e., quadrotor *i* does not communicate with any other quadrotors at time *k*.

### 4.2. Trajectory Planning Based on MPC and DCBF

Cooperative trajectory planning for quadrotor swarms is a multi-variable and multi-constraint optimization problem. Combining MPC and DCBF, we aim to generate a safe and smooth trajectory under collision avoidance and dynamics constraints. Consider a swarm system with *N* quadrotors in a shared workspace $W\in R^{3}$ . This finite-time optimization problem with constraints at time *k* within a prediction horizon *H* can be formulated as follows:

(12a) $$\begin{array}{cc} \min_{v_{i}^{k:k+H-1|k},\;\omega_{i}^{0:H-1}} & \sum_{i=1}^{N}J_{i}^{k}\left(z_{i}^{k:k+H|k},v_{i}^{k:k+H-1|k}\right)+J_{i}^{k}\left(\omega_{i}^{0:H-1}\right) \end{array}$$

(12b) $$\begin{array}{cc} \;s.t.\;\; & z_{i}^{k+t+1|k}=Az_{i}^{k+t|k}+Bv_{i}^{k+t|k}, \end{array}$$

(12c) $$\begin{array}{cc} \; & \zeta_{i}^{k+t|k}=Cz_{i}^{k+t|k}, \end{array}$$

(12d) $$\begin{array}{cc} \; & z_{i}^{k+t|k}\in \Omega , \end{array}$$

(12e) $$\begin{array}{cc} \; & z_{i}^{k|k}=z_{i}^{k}, \end{array}$$

(12f) $$\begin{array}{cc} \; & DCBC_{ij}^{k+t|k}\geq 0,\;\forall j\in N_{i}, \end{array}$$

(12g) $$\begin{array}{cc} \; & DCBC_{io}^{k+t|k}\geq 0,\;\forall o\in O, \end{array}$$

(12h) $$\begin{array}{cc} \; & \forall t\in \{0,1,\ldots ,H-1\}, \end{array}$$

where *N* is the number of quadrotors, and k+t∣k denotes the prediction at time *k* for the state at time k+t . The cost function (12a) is composed of two parts along the prediction horizon *H*, i.e., the cost $J_{i}^{k}\left(z_{i}^{k:k+H|k},v_{i}^{k:k+H-1|k}\right)$ with respect to the variables $z_{i}^{k:k+H|k}$ and $v_{i}^{k:k+H-1|k}$ , and the additional cost $J_{i}^{k}\left(\omega_{i}^{0:H-1}\right)$ with respect to the slack variables $\omega_{i}^{0:H-1}$ . (12b) and (12c) are the linear flat model of the quadrotor described by (6), while (12d) and (12e) denote the system dynamics constraints and initial state conditions, respectively. To ensure the safety of the trajectory, (12f) and (12g) give two types of DCBF constraints defined by (10). The specific forms of the cost function and constraints in the optimization are described in detail below.

(1) *Cost* $J_{i}^{k}\left(z_{i}^{k:k+H|k},v_{i}^{k:k+H-1|k}\right)$ : The cost function consists of three parts, including the terminal cost $p(z_{i}^{k+H|k})$ , the stage cost $q(z_{i}^{k+t|k},v_{i}^{k+t|k})$ , and the input change rate cost $r(v_{i}^{k+t|k},v_{i}^{k+t-1|k})$ , as shown below:
(13) $$\begin{array}{c} J_{i}^{k}\left(z_{i}^{k:k+H|k},v_{i}^{k:k+H-1|k}\right)=p\left(z_{i}^{k+H|k}\right)+\sum_{t=0}^{H-1}q\left(z_{i}^{k+t|k},v_{i}^{k+t|k}\right)+\sum_{t=1}^{H-1}r\left(v_{i}^{k+t|k},v_{i}^{k+t-1|k}\right), \end{array}$$
(14) $$p\left(z_{i}^{k+H|k}\right)={∥z_{i}^{k+H|k}-z_{i}^{d}∥}_{P}^{2},$$
(15) $$q\left(z_{i}^{k+t|k},v_{i}^{k+t|k}\right)=∥z_{i}^{k+t|k}-z_{i}^{d}{∥}_{Q}^{2}+{∥v_{i}^{k+t|k}∥}_{R}^{2},$$
(16) $$r\left(v_{i}^{k+t|k},v_{i}^{k+t-1|k}\right)={∥v_{i}^{k+t|k}-v_{i}^{k+t-1|k}∥}_{S}^{2},$$
  where *P*, *Q*, *R*, and *S* denote the weight matrices of the corresponding parts, respectively. $z_{i}^{d}$ is the target state of quadrotor *i*. From Equations (14)–(16), it can be seen that this cost penalizes the deviation of the predicted state from the target state, the size of the input, and the size of the input variations along the prediction horizon *H*. Therefore, the objective of the optimization problem is to enable the quadrotors to rapidly approach the target state while minimizing input size and its variation.
(2) *Cost* $J_{i}^{k}\left(\omega_{i}^{0:H-1}\right)$ : The additional cost function $J_{i}^{k}\left(\omega_{i}^{0:H-1}\right)$ is to drive the slack variables $\omega_{i}^{0:H-1}=\left[\omega_{ij}^{0:H-1},\omega_{io}^{0:H-1}\right]$ close to 0 to ensure the safety of the generated trajectories as follows:
(17) $$J_{i}^{k}\left(\omega_{i}^{0:H-1}\right)=\sum_{t=0}^{H-1}\alpha {\left(\omega_{ij}^{t}\right)}^{2}+\alpha {\left(\omega_{io}^{t}\right)}^{2},$$
  where α is a weighting coefficient. It is advisable to set α to a large value to prevent excessive relaxation of the DCBF constraints, which is also verified in the simulation experiments. In addition, the cost function $J_{i}^{k}\left(\omega_{i}^{0:H-1}\right)$ can be tuned for different performance.
(3) *DCBF Constraints*: To generate collision-free trajectories, (12f) and (12g) provide safety constraints $DCBC_{ij}^{k+t|k}$ and $DCBC_{io}^{k+t|k}$ in the optimization problem to ensure the forward invariance of the corresponding safety set C . The formulation is as follows:
(18) $$\begin{array}{cc} DCBC_{ij}^{k+t|k} & ≜h_{ij}\left(\zeta_{i}^{k+t+1|k},\zeta_{j}^{k+t+1|k}\right)-\left(1-\gamma_{ij}\right)h_{ij}\left(\zeta_{i}^{k+t|k},\zeta_{j}^{k+t|k}\right)+\omega_{ij}^{t}\geq 0,\;\forall j\in N_{i}, \end{array}$$
(19) $$\begin{array}{cc} DCBC_{io}^{k+t|k} & ≜h_{io}\left(\zeta_{i}^{k+t+1|k},p_{o}\right)-\left(1-\gamma_{io}\right)h_{io}\left(\zeta_{i}^{k+t|k},p_{o}\right)+\omega_{io}^{t}\geq 0,\;\forall o\in O. \end{array}$$

**Figure 3:**
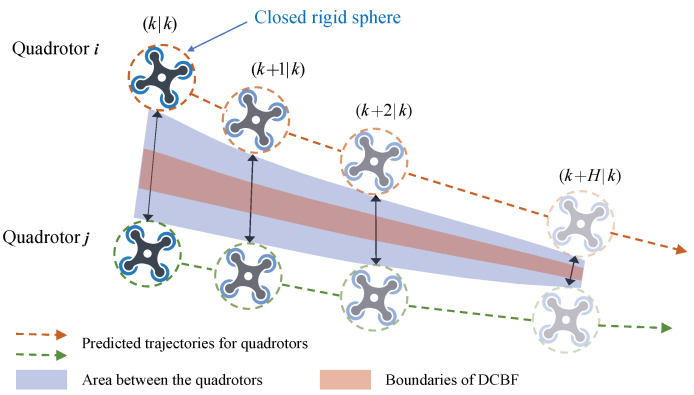
DCBF-based collision avoidance between quadrotor *i* and quadrotor *j*. The black solid arrows indicate the positions at the identical global time. The boundaries of the DCBF are defined by the corresponding constraints to prevent the quadrotors from approaching each other too fast.

We model each quadrotor *i* as a closed rigid sphere with radius $r_{i}$ and each obstacle *o* as a closed ellipsoid with semi-major axis $\left(a_{o},b_{o},c_{o}\right)$ . Similar to [18], the corresponding *h*-functions are given by

(20) $$h_{ij}\left(\zeta_{i}^{k+t|k},\zeta_{j}^{k+t|k}\right)=∥p_{i}^{k+t|k}-p_{j}^{k+t|k}∥-r_{i}-r_{j}\geq 0,$$

(21) $$h_{io}\left(\zeta_{i}^{k+t|k},p_{o}\right)={∥p_{i}^{k+t|k}-p_{o}∥}_{W}-1\geq 0,$$

where $p_{i}^{k+t|k}$ represents the position of quadrotor *i* and is a component of the output $\zeta_{i}^{k+t|k}$ . Correspondingly, $p_{o}$ is the position of obstacle *o*. (20) implies that the sphere representing quadrotor *i* does not intersect the sphere representing quadrotor *j*, see Figure 3 for details. Similarly, (21) can be interpreted as approximating the obstacle *o* as an enlarged ellipsoid to check whether the position of quadrotor *i* is inside it [16], and $W=\mathrm{diag}(1/{\left(a_{o}+r_{i}\right)}^{2},1/{\left(b_{o}+r_{i}\right)}^{2},1/{\left(c_{o}+r_{i}\right)}^{2})$ . Since the DCBF is nonconvex, the real-time computation of problem (12) is challenging when it has a large prediction horizon *H*. Therefore, we linearize the DCBF around the result of the previous iteration at each time step using a first-order Taylor expansion as follows:

(22) $$\begin{array}{cc} \; & h_{ij}\left(\zeta_{i}^{k+t|k},\zeta_{j}^{k+t|k}\right)=\eta_{ij}^{T}\left(p_{i}^{k+t|k}-p_{j}^{k+t|k}\right)-r_{i}-r_{j}\geq 0,\\ \; & h_{io}\left(\zeta_{i}^{k+t|k},p_{o}\right)=\eta_{io}^{T}W\left(p_{i}^{k+t|k}-p_{o}\right)-1\geq 0,\\ \; & \eta_{ij}=\frac{{\hat{p}}_{i}^{k+t|k}-{\hat{p}}_{j}^{k+t|k}}{∥{\hat{p}}_{i}^{k+t|k}-{\hat{p}}_{j}^{k+t|k}∥},\;\eta_{io}=\frac{{\hat{p}}_{i}^{k+t|k}-p_{o}}{∥{\hat{p}}_{i}^{k+t|k}-p_{o}∥}, \end{array}$$

where ${\hat{p}}_{i}^{k+t|k}$ is the result of the previous iteration for quadrotor *i*. Then, we can solve problem (12) with an iterative convex optimization. The detailed steps of the solution are described in the following section.

**Remark** **1.** *In this paper, we consider that the yaw direction of the quadrotor is fixed as ψ=0 in the desired trajectory, and the focus is on the spatial position of the quadrotor.*

The above is the optimization problem for trajectory planning. We will introduce a fully distributed trajectory planning algorithm for efficiently computing future trajectories for all quadrotors in a swarm, as explained in the following section.

## 5. DMPC-ADMM Based Trajectory Planning

In this section, we reformulate the trajectory planning (12) for quadrotor swarms based on ADMM, converting it into a fully distributed framework. This approach decomposes the original high-dimensional optimization problem into *N* low-dimensional optimization subproblems, allowing each quadrotor *i* to compute its optimal trajectory in parallel to speed up the online solution of the problem. The focus lies in determining the communication information and mode to coordinate the quadrotors in order to avoid collision among them.

### 5.1. General ADMM Formulation

Here, we present a brief overview of ADMM. For more details, the readers can refer to [32]. ADMM is an iterative algorithm for solving distributed optimization problems. It decomposes the original problem into several subproblems and solves them by updating the multipliers and alternating iterations. The standard ADMM considers the following optimization problem with equation constraints.

(23) $$\min_{x,z}f\left(x\right)+g\left(z\right)\;\;s.t.\;\;Ax+Bz=c,$$

where $x\in R^{n1}$ and $z\in R^{n2}$ are the original decision variables. $f:R^{n_{1}}\to R$ , $g:R^{n_{2}}\to R$ , $A\in R^{n_{3}\times n_{1}}$ , $B\in R^{n_{3}\times n_{2}}$ , and $c\in R^{n_{3}}$ . ADMM utilizes the augmented Lagrangian function with an additional quadratic penalty term to obtain better convergence. (24) is the augmented Lagrangian function of problem (23). The steps of ADMM iteratively solving (23) are described in Algorithm 1 until the predefined stopping iteration condition is satisfied.

(24) $$L_{\rho }\left(x,z,\lambda \right)=f\left(x\right)+g\left(z\right)+\lambda^{T}\left(Ax+Bz-c\right)+\frac{\rho }{2}{∥Ax+Bz-c∥}_{2}^{2},$$

where $\lambda \in R^{n_{3}}$ is the Lagrange multiplier and ρ>0 is the penalty coefficient. Although ρ is independent of the convergence of the optimization problem (23), it affects the convergence rate. And we can obtain a suitable ρ by sufficient experiments. The next subsection describes how to reformulate the trajectory planning problem (12) as an adaptation of the structure of problem (23) to solve it in a distributed manner.

### 5.2. Problem Decomposition Based on ADMM

For trajectory planning of quadrotor swarms, the only coupling among quadrotors is represented by the collision avoidance constraints (18). The purpose of interactions among quadrotors is to tell the neighbors about their own future trajectories so that they can avoid collision in advance. In such scenarios, quadrotors need to agree on safe trajectories within the confined space. Motivated by [36], each quadrotor maintains communication with its neighbors while computing trajectories by introducing duplicates of its neighbors’ trajectories. Specifically, we use $w_{i}$ to represent the duplicate of the trajectory $\zeta_{i}$ , and $w_{i\to j}$ is the duplicate of quadrotor *i* for the trajectory $\zeta_{j}$ of quadrotor *j*, which can also be interpreted as the desired trajectory proposed by quadrotor *i* for quadrotor *j*. Then the control barrier function $h_{ij}\left(\zeta_{i}^{k+t|k},\zeta_{j}^{k+t|k}\right)\geq 0$ can be reformulated as

(25) $$\begin{array}{cc} \; & {\bar{h}}_{ij}\left(w_{i}^{k+t|k},w_{i\to j}^{k+t|k}\right)\geq 0,\\ \; & w_{i}^{k+t|k}=\zeta_{i}^{k+t|k},\;w_{i\to j}^{k+t|k}=\zeta_{j}^{k+t|k},\;\forall j\in N_{i}. \end{array}$$

It can be seen that using $w_{i}$ and $w_{i\to j}$ , the coupling among quadrotors is successfully decoupled. Here, we use I to denote an indicator function, defined as

(26) $$I_{A}\left(x\right):=\left\{\begin{array}{cc} 0 & \;\mathrm{if}\;x\in A,\\ \infty & \;\mathrm{otherwise}.\; \end{array}\right.$$

Compared to the problem (23), we replace the cost function of the optimization problem (12) with $f\left(z_{i}^{k:k+H|k},v_{i}^{k:k+H-1|k},\omega_{i}^{0:H-1}\right)+g(w_{i}^{k:k+H|k},w_{i\to j}^{k:k+H|k})$ as follows:

(27) $$\begin{array}{cc} f(z_{i}^{k:k+H|k},v_{i}^{k:k+H-1|k},\omega_{i}^{0:H-1})\; & =\sum_{i=1}^{N}[J_{i}^{k}\left(z_{i}^{k:k+H|k},v_{i}^{k:k+H-1|k}\right)+J_{i}^{k}\left(\omega_{i}^{0:H-1}\right)\\ \; & \;+\;I_{\Phi }\left(z_{i}^{k:k+H|k},v_{i}^{k:k+H-1|k}\right)], \end{array}$$

(28) $$g\left(w_{i}^{k:k+H|k},w_{i\to j}^{k:k+H|k}\right)=\sum_{i=1}^{N}\left[\sum_{j\in N_{i}}I_{DCBC_{ij}\geq 0}\left(w_{i}^{k:k+H|k},w_{i\to j}^{k:k+H|k}\right)\right],$$

where the set Φ denotes all the constraints in (12) except for inter-quadrotor collision avoidance. So, the optimization problem (12) can be transformed into the form as follows:

(29) $$\begin{array}{cc} \min_{v_{i}^{k:k+H-1|k},\;w_{i}^{k:k+H|k},\;w_{i\to j}^{k:k+H|k},\;\omega_{i}^{0:H-1}} & f\left(z_{i}^{k:k+H|k},v_{i}^{k:k+H-1|k},\omega_{i}^{0:H-1}\right)+g(w_{i}^{k:k+H|k},w_{i\to j}^{k:k+H|k})\\ s.t.\;\; & w_{i}^{k+t|k}=\zeta_{i}^{k+t|k},\;w_{i\to j}^{k+t|k}=\zeta_{j}^{k+t|k},\\ \; & \forall t\in \left\{0,1,\ldots ,H-1\right\},\;\forall j\in N_{i}. \end{array}$$

Obviously, (29) is an optimization problem with equation constraints, whose augmented Lagrangian form is given in (30), where $\lambda_{i}$ and $\lambda_{i\to j}$ are the corresponding dual variables and ρ is the penalty coefficient. This allows us to solve this optimization problem according to Algorithm 1, iteratively updating $z_{i}^{k:k+H|k},v_{i}^{k:k+H-1|k},\omega_{i}^{0:H-1},w_{i}^{k:k+H|k}$ , $w_{i\to j}^{k:k+H|k}$ , $\lambda_{i}^{k:k+H|k}$ , and $\lambda_{i\to j}^{k:k+H|k}$ sequentially until the stopping condition is satisfied or the maximum number of iterations is reached. Each quadrotor *i* computes its trajectory while maintaining communication with its neighbors to exchange future trajectories of the prediction horizon *H*.

(30) $$\begin{array}{cc} L_{\rho }= & f\left(z_{i}^{k:k+H|k},v_{i}^{k:k+H-1|k},\omega_{i}^{0:H-1}\right)+g(w_{i}^{k:k+H|k},w_{i\to j}^{k:k+H|k})+\sum_{i=1}^{N}\left(M_{i}+\sum_{j\in N_{i}}M_{i\to j}\right)\\ = & \sum_{i=1}^{N}[L_{\rho ,i}\left(\zeta_{i}^{k+H|k},z_{i}^{k:k+H|k},v_{i}^{k:k+H-1|k},\omega_{i}^{0:H-1},w_{i}^{k:k+H|k},\lambda_{i}^{k:k+H|k}\right)\\ \; & +\sum_{j\in N_{i}}L_{\rho ,i\to j}\left(\zeta_{j}^{k+H|k},w_{i\to j}^{k:k+H|k},\lambda_{i\to j}^{k:k+H|k}\right)]\\ = & \sum_{i=1}^{N}[L_{\rho ,i}\left(\zeta_{i}^{k:k+H|k},z_{i}^{k:k+H|k},v_{i}^{k:k+H-1|k},\omega_{i}^{0:H-1},w_{i}^{k:k+H|k},\lambda_{i}^{k:k+H|k}\right)\\ \; & +\sum_{j\in N_{i}}L_{\rho ,j\to i}\left(\zeta_{i}^{k:k+H|k},w_{j\to i}^{k:k+H|k},\lambda_{j\to i}^{k:k+H|k}\right)] \end{array}$$

**Algorithm 1** ADMM.

$\begin{array}{cc} \mathrm{repeat} & \;1:\;x\leftarrow \arg\min_{x}L_{\rho }\left(x,z,\lambda \right)\\ & \;2:\;z\leftarrow \arg\min_{z}L_{\rho }\left(x,z,\lambda \right)\\ & \;3:\;\lambda \leftarrow \lambda +\rho \frac{\partial }{\partial \lambda }L_{\rho }\left(x,z,\lambda \right) \end{array}$

$\begin{array}{c} \mathrm{until}\;\;\;\mathrm{satisfaction}\;\mathrm{of}\;a\;\mathrm{stopping}\;\mathrm{criterion} \end{array}$




The last equation in (30) holds because a bi-directional interaction is assumed, i.e., $j\in N_{i}\Leftrightarrow i\in N_{j}$ . So, we can flip the indexes *i* and *j*. $M_{i}$ and $M_{i\to j}$ in (30) are defined as follows:

(31) $$\begin{array}{cc} \; & M_{i}=\sum_{t=0}^{H}\lambda_{i}^{T}\left(\zeta_{i}^{k+t|k}-w_{i}^{k+t|k}\right)+\frac{\rho }{2}{∥\zeta_{i}^{k+t|k}-w_{i}^{k+t|k}∥}_{2}^{2},\\ \; & M_{i\to j}=\sum_{t=0}^{H}\lambda_{i\to j}^{T}\left(\zeta_{j}^{k+t|k}-w_{i\to j}^{k+t|k}\right)+\frac{\rho }{2}{∥\zeta_{j}^{k+t|k}-w_{i\to j}^{k+t|k}∥}_{2}^{2} \end{array}$$

According to Algorithm 1, we separate the Lagrangian function (30) into two subproblems. We first minimize $L_{\rho }$ over the local variables $v_{i}^{k:k+H-1|k}$ and $\omega_{i}^{0:H-1}$ , and then minimize it over the global variables $w_{i}^{k:k+H|k}$ and ${\left\{w_{i\to j}^{k:k+H|k}\right\}}_{j\in N_{i}}$ . Algorithm 2 describes the steps of the distributed trajectory planning algorithm based on DMPC-ADMM, containing the steps of prediction, coordination, and mediation, as well as two communications, all of which are executed iteratively on the quadrotors in parallel. The prediction step ensures that each quadrotor *i* computes a trajectory $\zeta_{i}^{k:k+H|k}$ of prediction horizon *H* under the constraints represented by Φ . The trajectory should be close to the target position while not deviating too much from its collision-free trajectory $w_{i}^{k:k+H|k}$ and the desired trajectories ${\left\{w_{j\to i}^{k:k+H|k}\right\}}_{j\in N_{i}}$ that its neighbors propose. Each quadrotor *i* then exchanges the updated trajectory $\zeta_{i}^{k:k+H|k}$ with its neighbors in the first round of communication. In the coordination step, each quadrotor *i* updates $\left(w_{i}^{k:k+H|k},{\left\{w_{i\to j}^{k:k+H|k}\right\}}_{j\in N_{i}}\right)$ . This is done by coordinating with its neighbors to avoid collision while staying as close as possible to the trajectory $\zeta_{i}^{k:k+H|k}$ computed in the prediction step. In the mediation step, the Lagrange multipliers $\left(\lambda_{i}^{k:k+H|k},{\left\{\lambda_{i\to j}^{k:k+H|k}\right\}}_{j\in N_{i}}\right)$ that accumulate the deviation between the trajectories computed in the prediction step and the coordination step are updated, further facilitating the quadrotors to reach a consensus on the trajectories of prediction horizon *H*. Finally, the desired Lagrange multipliers ${\left\{\lambda_{i\to j}^{k:k+H|k}\right\}}_{j\in N_{i}}$ and trajectories ${\left\{w_{i\to j}^{k:k+H|k}\right\}}_{j\in N_{i}}$ are exchanged with its neighbors in the second round of communication. The iteration is finished when the stopping iteration condition (35) is satisfied or the maximum number of iterations $l_{max}$ is reached. The first control input $v_{i}^{k}$ is selected from $v_{i}^{k:k+H|k}$ to guide the controller at time k+1 . Then, this algorithm repeats in this manner until all the quadrotors have reached the target positions.
**Algorithm 2** Distributed algorithm based on DMPC-ADMM.
1:
$\forall i\in \left\{1,\ldots ,N\right\},\;\mathrm{Initialize}z_{i}^{0},k=0$ .2:**while** target positions not reached **do**3:    **for** ∀i∈{1,…,N} in parallel **do**4:       Initialize $\lambda_{i}^{k:k+H|k},w_{i}^{k:k+H|k},{\left\{\lambda_{i\to j}^{k:k+H|k},w_{i\to j}^{k:k+H|k}\right\}}_{j\in N_{i}}$ .5:       Set l=0 and update $N_{i}$ .6:       **while** $l<l_{\max}$ or stopping criterion (35) is not satisfied **do**7:           **1. prediction:** update $\zeta_{i}^{k:k+H|k}$ with

(32)
$$\begin{array}{cc} \underset{v_{i}^{k:k+H|k},\;\omega_{i}^{0:H-1}}{\arg\min} & L_{\rho ,i}\left(\zeta_{i}^{k:k+H|k},z_{i}^{k:k+H|k},v_{i}^{k:k+H-1|k},\omega_{i}^{0:H-1},w_{i}^{k:k+H|k},\lambda_{i}^{k:k+H|k}\right)\;\\ & +\sum_{j\in N_{i}}L_{\rho ,j\to i}\left(\zeta_{i}^{k:k+H|k},w_{j\to i}^{k+t|k},\lambda_{j\to i}^{k:k+H|k}\right) \end{array}$$
8:           **2. communication 1:**9:              send $\zeta_{i}^{k:k+H|k}$ to $j\in N_{i}$ ;10:            receive $\left\{\zeta_{j}^{k:k+H|k}\right\}$ from $j\in N_{i}$ .11:         **3. coordination:**12:            update $\left(w_{i}^{k:k+H|k},{\left\{w_{i\to j}^{k:k+H|k}\right\}}_{j\in N_{i}}\right)$ with

(33)
$$\begin{array}{cc} \underset{w_{i}^{k:k+H|k},\;w_{i\to j}^{k:k+H|k}}{\arg\min} & L_{\rho ,i}\left(\zeta_{i}^{k:k+H|k},z_{i}^{k:k+H|k},v_{i}^{k:k+H-1|k},\omega_{i}^{0:H-1},w_{i}^{k:k+H|k},\lambda_{i}^{k:k+H|k}\right)\;\\ & +\sum_{j\in N_{i}}L_{\rho ,i\to j}\left(\zeta_{j}^{k:k+H|k},w_{i\to j}^{k:k+H|k},\lambda_{i\to j}^{k:k+H|k}\right) \end{array}$$
13:         **4. mediation:**14:            update $\left(\lambda_{i}^{k:k+H|k},{\left\{\lambda_{i\to j}^{k:k+H|k}\right\}}_{j\in N_{i}}\right)$ with

$$\begin{array}{c} \lambda_{i}^{k:k+H|k}\leftarrow \lambda_{i}^{k:k+H|k}+\rho \left(\zeta_{i}^{k:k+H|k}-w_{i}^{k:k+H|k}\right),\;\\ \lambda_{i\to j}^{k:k+H|k}\leftarrow \lambda_{i\to j}^{k:k+H|k}+\rho \left(\zeta_{j}^{k:k+H|k}-w_{i\to j}^{k:k+H|k}\right),\;\forall j\in N_{i}. \end{array}$$
15:         **5. communication 2:**16:            send $\left(\lambda_{i\to j}^{k:k+H|k},w_{i\to j}^{k:k+H|k}\right)$ to $j\in N_{i}$ ;17:            receive $\left\{\lambda_{j\to i}^{k:k+H|k},w_{j\to i}^{k:k+H|k}\right\}$ from $j\in N_{i}$ .18:     **end while**19:     Select the first control input $v_{i}^{k}$ from $v_{i}^{k:k+H-1|k}$ .20:     Update state $z^{k+1}=Az^{k}+Bv^{k},\;\zeta_{i}^{k+1}=Cz_{i}^{k+1}$ .21:  **end for**22:  
k←k+1andl←l+1 .23:**end while**


In (22), we give a specific formulation of the linearized DCBF such that (29) is a convex problem with constraints. Here, we solve (32) and (33) in an iterative manner to approximate the optimal solution; see Algorithms 3 and 4. In Algorithm 3, the updated output ${\bar{\zeta }}_{i,d}^{k:k+H|k}$ is passed between iterations allowing for the linearization of $DCBC_{io}$ . The iteration is finished when the convergence criterion (34) is satisfied or the maximum number of iterations $d_{max}$ is reached. The updated optimal $\zeta_{i}^{k:k+H|k}$ is then exchanged with its neighbors in the first round of communication. Algorithm 4 follows the same principle as Algorithm 3, so it is not explained further here.

(34) $$∥\zeta_{i,d}^{*,k:k+H|k}-{\bar{\zeta }}_{i,d}^{k:k+H|k}∥\leq \epsilon_{abs},$$

where $\epsilon_{abs}>0$ is constant, (34) implies that the iteration of Algorithm 3 is finished when the absolute value of the change in output ${\bar{\zeta }}_{i,d}^{k:k+H|k}$ is less than $\epsilon_{abs}$ .
**Algorithm 3** Iterative convex optimization of (32).
1:Set initial guess ${\bar{\zeta }}_{i,0}^{k:k+H|k}$ .2:**Initialize** d=0 .3:**while** $d<d_{\max}$ or convergence criteria (34) is not satisfied **do**4:     Linearize safety constraints $DCBC_{io}$ (12g) with ${\bar{\zeta }}_{i,d}^{k:k+H|k}$ .5:     Solve a convex optimization problem with constraints and obtain the optimal value of state $\zeta_{i,d}^{*,k:k+H|k}$ .6:    Update ${\bar{\zeta }}_{i,d+1}^{k:k+H|k}=\zeta_{i,d}^{*,k:k+H|k}$ .7:    
d←d+1.
8:**end while**9:Update $\zeta_{i}^{k:k+H|k}$ = $\zeta_{i,d}^{*,k:k+H|k}$ .

**Algorithm 4** Iterative convex optimization of (33).
1:Set initial guess ${\bar{w}}_{i,0}^{k:k+H|k}$ , ${\bar{w}}_{i\to j,0}^{k:k+H|k}$ .2:**Initialize** d=0 .3:**while** $d<d_{\max}$ or convergence criteria is not satisfied **do**4:     Linearize safety constraints $DCBC_{ij}$ (12f) with ${\bar{w}}_{i,d}^{k:k+H|k}$ , ${\bar{w}}_{i\to j,d}^{k:k+H|k}$ .5:     Solve a convex optimization problem with constraints and obtain the optimal value of state $w_{i,d}^{*,k:k+H|k}$ , $w_{i\to j,d}^{*,k:k+H|k}$ .6:    Update ${\bar{w}}_{i,d+1}^{k:k+H|k}=w_{i,d}^{*,k:k+H|k}$ , ${\bar{w}}_{i\to j,d+1}^{k:k+H|k}=w_{i\to j,d}^{*,k:k+H|k}$ .7:    
d←d+1.
8:**end while**9:Update $w_{i}^{k:k+H|k}=w_{i,d}^{*,k:k+H|k}$ , $w_{i\to j}^{k:k+H|k}=w_{i\to j,d}^{*,k:k+H|k}$ .

**Remark** **2.** *For the initialization of Algorithm 2, we use the optimal result of the previous loop of DMPC-ADMM. Likewise, we use the optimal result of the previous iteration as the initial guess in Algorithms 3 and 4. This hot-start strategy can speed up the convergence of the algorithm, especially in slowly changing scenarios.*

Each quadrotor *i* plans its trajectory $\zeta_{i}^{k:k+H|k}$ by taking into account its optimal trajectory $w_{i}^{k:k+H|k}$ and the desired trajectories ${\left\{w_{j\to i}^{k:k+H|k}\right\}}_{j\in N_{i}}$ that are proposed by its neighbors. Thus, the stopping iteration condition for each MPC-ADMM loop can be designed in the following form:

(35) $$\begin{array}{cc} \; & ∥\zeta_{i}^{k:k+H|k}-w_{i}^{k:k+H|k}∥\leq \epsilon_{1},\\ \; & ∥\zeta_{i}^{k:k+H|k}-\frac{1}{num_{i}^{nbrs}}\sum_{j\in N_{i}}w_{j\to i}^{k:k+H|k}∥\leq \epsilon_{2}, \end{array}$$

where $\epsilon_{1}$ and $\epsilon_{2}$ represent tolerable deviation thresholds, respectively, and $num_{i}^{nbrs}$ is the number of neighbors of quadrotor *i*. With sufficient iterations, the quadrotor *i* will agree with its neighbors on the trajectories of prediction horizon *H*. However, extensive simulations show that the algorithm reaches an accepted result after a certain number of iterations. To reduce the computational burden, we present an empirical value of $l_{max}$ that allows us to compute a sub-optimal result.

### 5.3. Event-Triggered Mechanism

**Figure 4:**
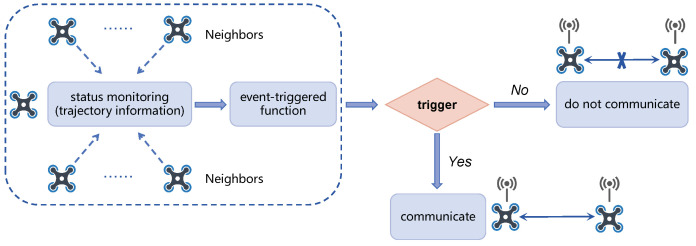
Structure of the event detector.

In general, the maximum communicable distance $\Delta^{dect}$ is much greater than the distance that quadrotors can fly within the prediction horizon *H*. Consequently, Algorithm 2 exhibits substantial redundancy in inter-quadrotor communication, especially in scenarios involving many quadrotors. Inspired by [39,40], we design an event-triggered mechanism that initiates communication only when a specific trigger condition is met. The critical element of the event-triggered mechanism lies in the design of the event detector. The content and operational mode of the event detector determines the functioning of the event-triggered mechanism, subsequently influencing the communication frequency of the system, as shown in Figure 4.

Considering a scenario in which two quadrotors are widely separated, there is no risk of collision within the prediction horizon *H*, so they need not maintain communication while generating trajectories. Within the distributed framework, at time *k*, quadrotor *i* listens to the position of its neighbor $j\in N_{i}$ to determine whether the event is triggered. We design the trigger function of the event detector as follows:

(36) $$E\left(p_{i}^{k},p_{j}^{k}\right)=∥p_{i}^{k}-p_{j}^{k}∥-2HT_{s}v_{\max}-r_{i}-r_{j},\;j\in N_{i},$$

where $p_{i}^{k}$ , $p_{j}^{k}$ are the positions of quadrotor *i* and its neighbor $j\in N_{i}$ at time *k*, $T_{s}$ is the time step, and $v_{max}$ is the maximum velocity of the quadrotor. The trigger condition holds when the value of the trigger function (36) is negative. We then incorporate this event-triggered mechanism into Algorithm 2, which reduces communication resources and the number of collision avoidance constraints $DCBC_{ij}\geq 0$ .

**Remark** **3.** *The optimization problem may become infeasible when the flight space of the quadrotors is highly competitive. In this case, we can slow down the quadrotors quickly, and after a few time steps, the problem becomes feasible again.*

## 6. Simulation Experiments and Results

In this section, we describe and evaluate the implementation of our method in simulation experiments. The simulation results validate the efficiency of our method. And we use the RflySim platform [41] to validate the proposed method. The platform provides quadrotor dynamics that are almost indistinguishable from actual scenario flights. The trajectories of all quadrotors are generated by our method, and the PID controller is used to track the trajectories. A video demonstration on the RflySim platform can be found at https://www.bilibili.com/video/BV11u4y1w7HU (accessed on 15 January 2024).

### 6.1. Experimental Setup

All the quadrotors have the same dynamics model, and we apply a box constraint set Ω on the flat state as is done in [42] for trajectory generation, as shown in (37). The parameters used in the simulation are shown in Table 2. Here, we define two scenarios, one for obstacle avoidance flight in complex environments (Scenario 1) and one for exchanging positions flight (Scenario 2). We provide a performance comparison of our method with centralized MPC (CMPC), constant velocity MPC (CVMPC, treat the quadrotor as a constant velocity model), and distributed MPC (DMPC, use the future trajectories computed by its neighbors at the previous time as collision avoidance constraints) [16] for trajectory planning. The only difference among these methods is the coordination strategies, where all parameters are identical. All methods are solved using OSQP [43] with the modeling language Yalmip [44]. We use a Windows desktop with Intel Core i7-11700K (CPU 3.6 GHz) running MATLAB for all computations. In the simulation, we assume that the environmental information is known, the communication packets are not lost, and there are no external perturbations.

(37) $$\Omega =\{z\in R^{10}|-3\leq \dot{x},\dot{y},\dot{z}\leq 3;-1\leq \ddot{x},\ddot{y},\ddot{z}\leq 1;\psi \in \left[-\pi ,\pi \right]\}.$$

### 6.2. Performance Comparison of Different Methods

**Figure 5:**
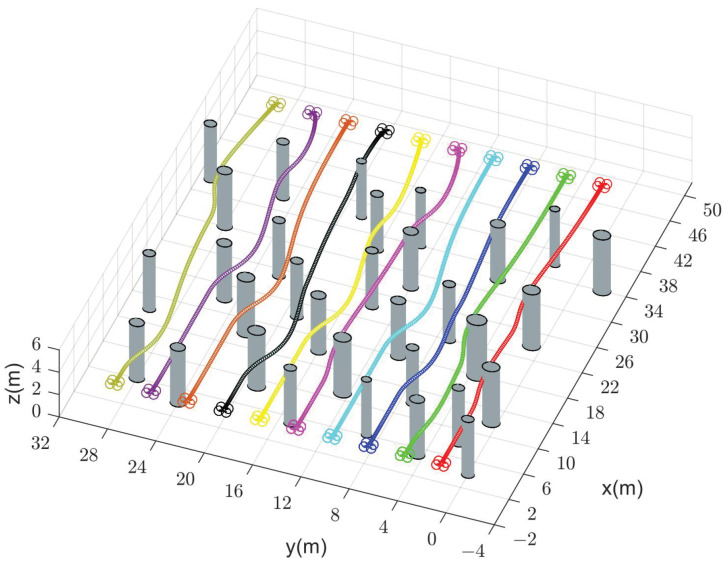
Generated trajectories of ten quadrotors (solid dots in different colors) cross a finite space with obstacles using our method (DMPC-ADMM).

**Figure 6:**
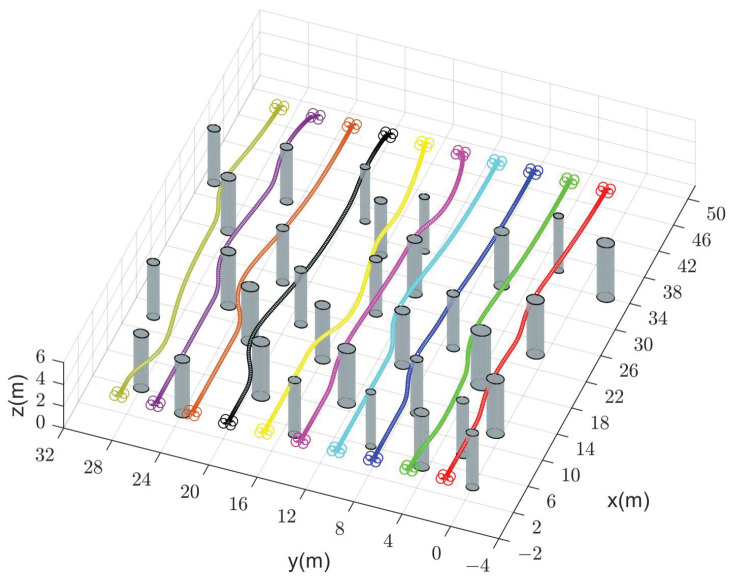
Generated trajectories of ten quadrotors (solid dots in different colors) cross a finite space with obstacles using CMPC.

**Figure 7:**
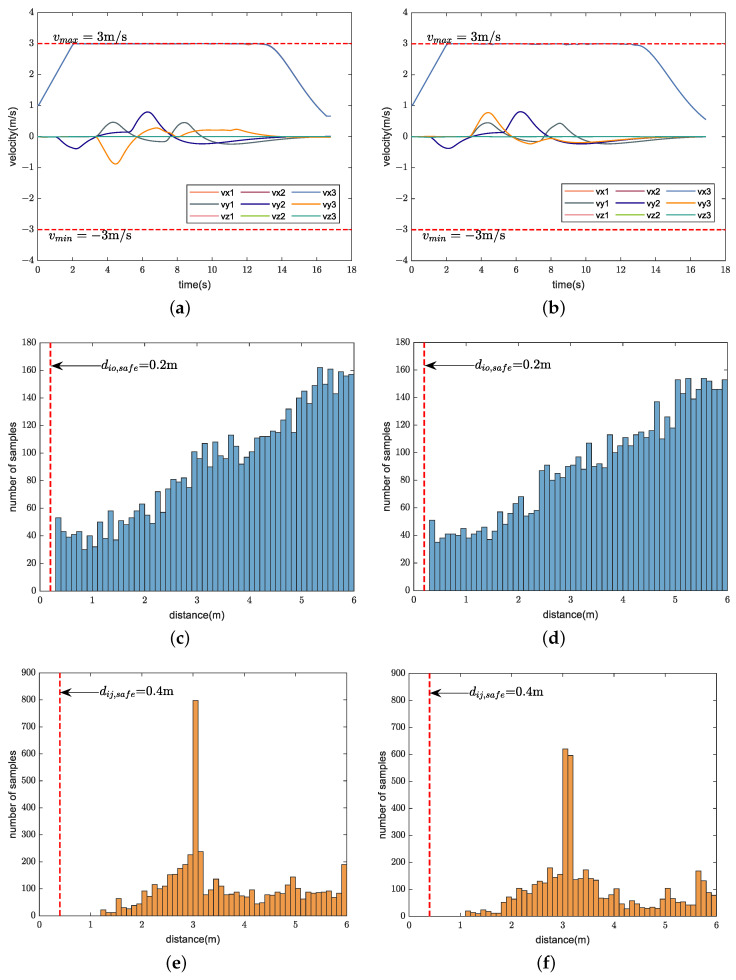
Simulation results of ten quadrotors cross a finite space with obstacles using our method (**left**) and CMPC (**right**). (**a**,**b**) Velocity variations. (**c**,**d**) Distance between quadrotors and obstacles (Only distance statistics within 6 m are shown). (**e**,**f**) Distance among quadrotors (Only distance statistics within 6 m are shown). The red dashed lines represent the maximum velocity $v_{max}=3\;m/s$ , the minimum velocity $v_{min}=-3\;m/s$ , the safe distance between quadrotors and obstacles $d_{io,safe}=0.2\;m$ , and the safe distance among quadrotors $d_{ij,safe}=0.4\;m$ , respectively.

**Figure 8:**
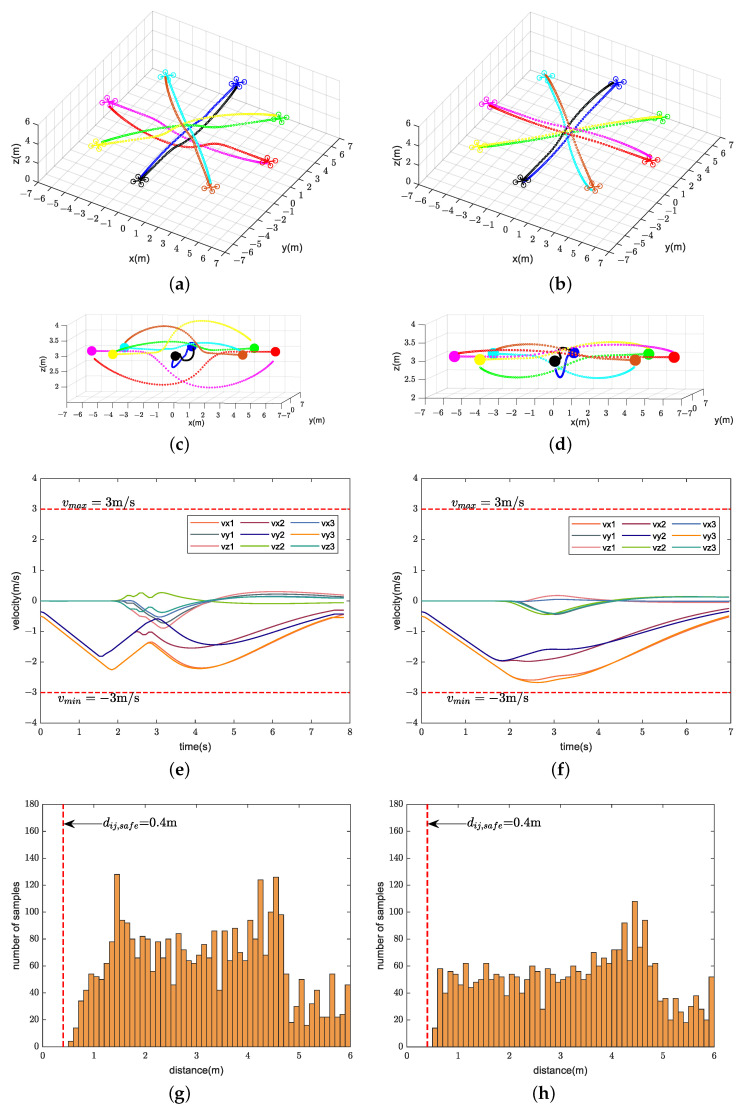
Simulation results of eight quadrotors (solid dots in different colors) exchanging positions flight using our method (**left**) and CMPC (**right**). (**a**,**b**) Overall view of the trajectories. (**c**,**d**) Side view of the trajectories. (**e**,**f**) Velocity variations. (**g**,**h**) Distance among quadrotors (Only distance statistics within 6 m are shown). The red dashed lines represent the maximum velocity $v_{max}=3\;m/s$ , the minimum velocity $v_{min}=-3\;m/s$ , and the safe distance among quadrotors $d_{ij,safe}=0.4\;m$ , respectively.

**Figure 9:**
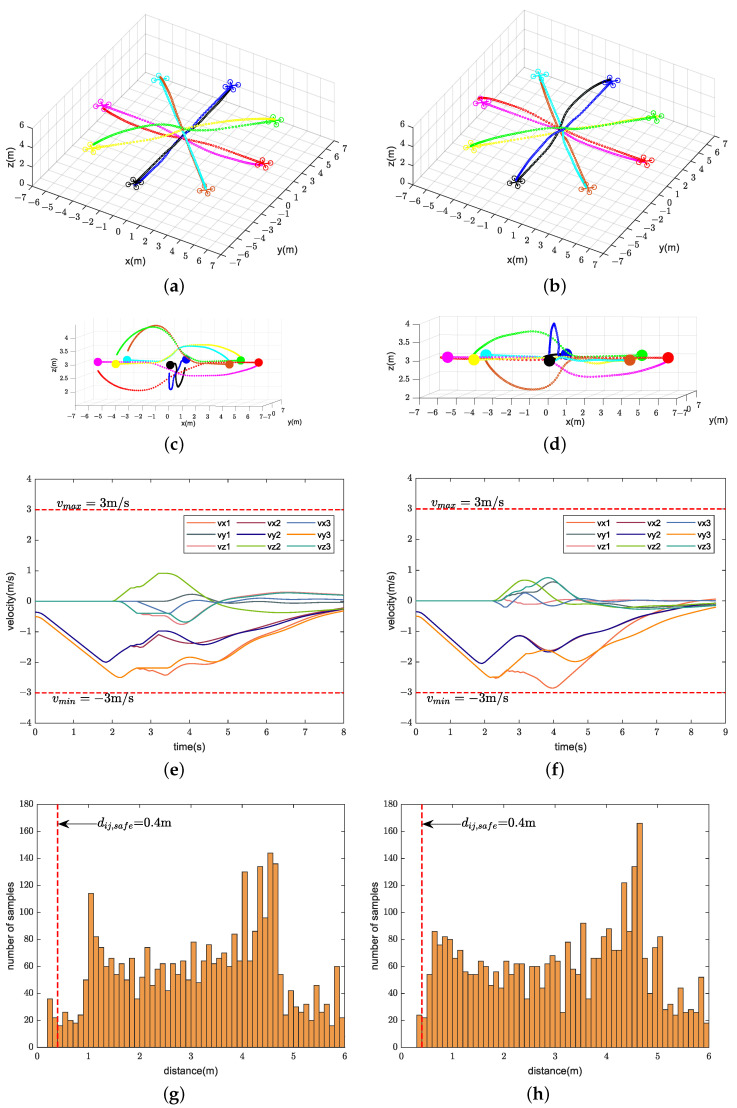
Simulation results of eight quadrotors (solid dots in different colors) exchanging positions flight using CVMPC (**left**) and DMPC (**right**). (**a**,**b**) Overall view of the trajectories. (**c**,**d**) Side view of the trajectories. (**e**,**f**) Velocity variations. (**g**,**h**) Distance among quadrotors (Only distance statistics within 6 m are shown). The red dashed lines represent the maximum velocity $v_{max}=3\;m/s$ , the minimum velocity $v_{min}=-3\;m/s$ , and the safe distance among quadrotors $d_{ij,safe}=0.4\;m$ , respectively.

We compare the performance of our method with CMPC, CVMPC, and DMPC in two scenarios. For Scenario 1 with ten quadrotors, Figure 5, Figure 6 and Figure 7 show the simulation results using our method and CMPC, respectively. Figure 8 and Figure 9 show the simulation results of the eight quadrotors in Scenario 2 using the four methods. From the velocity variations of three of the quadrotors, we observe that our method generates smoother trajectories compared to CVMPC and DMPC, and the statistics of the distance among quadrotors show that our method is safer. Moreover, the performance of the trajectories generated by our method is not much different from that of CMPC.

For further comparison, we report the trajectory length (minimum, maximum, mean value, and standard deviation to compare cooperativeness), the minimum distance $d_{ij,min}$ among quadrotors, the minimum distance $d_{io,min}$ between quadrotors and obstacles, as well as the average computation time of the four methods in Table 3 and Table 4. It can be observed that CVMPC and DMPC have a minimum distance lower than the safe distance due to the lack of coordination and take more time than our method due to the need for a greater number of iterations. Instead, our method and CMPC can achieve safe navigation, and the computation time of our method is much less than that of CMPC.

**Figure 10:**
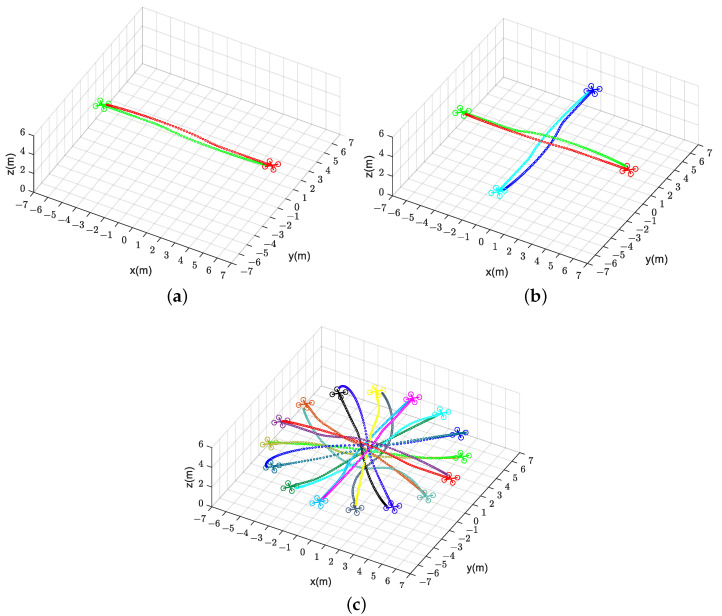
Generated trajectories of multiple quadrotors (solid dots in different colors) exchanging positions flight using our method (DMPC-ADMM). (**a**) Two quadrotors. (**b**) Four quadrotors. (**c**) Sixteen quadrotors.

In addition, Table 5 shows the comparison of the four methods in terms of average computation time, collision probability, and feasibility in Scenario 2. For each method, we generate trajectories of the quadrotors under 50 random initial and target states. We define the method as infeasible when the collision probability is greater than 50% . We observe that CMPC suffers from a large computational burden, while our method performs well at a much lower computational cost. The collision probability of our method and CMPC stays below 10% as the number of quadrotors increases. Instead, due to the deviation of trajectory information, CVMPC and DMPC become infeasible with a significant increase in collision probability, especially for CVMPC. Therefore, our method scales well with the number of quadrotors. Figure 10 illustrates the trajectories of two, four, and sixteen quadrotors for exchanging positions in flight.

### 6.3. Evaluation of Event-Triggered Mechanism

**Figure 11:**
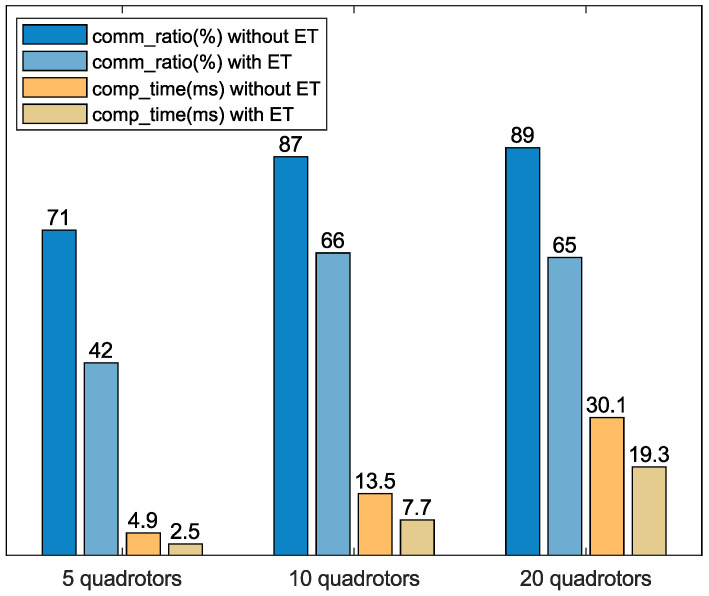
Comparison of communication ratio and computation time before and after adding the event-triggered mechanism.

To verify the effectiveness of the event-triggered mechanism, we compare the performance of the algorithm before and after adding the event-triggered mechanism under a uniform spatial distribution of different numbers of quadrotors in Scenario 1. Define a metric COMM as the communication cost and the communication ratio as the ratio of the communication cost after adding the event-triggered mechanism to the original communication cost, as shown in (38). Figure 11 shows a significant reduction in communication ratio and computation time after adding the event-triggered mechanism. Therefore, the event-triggered mechanism greatly improves the performance of the distributed algorithm.

(38) $$\begin{array}{cc} \; & COMM=\sum_{i=1}^{N}\sum_{j=1}^{N}comm_{ij},\\ \; & comm_{ij}=\left\{\begin{array}{cc} 1, & i\;\mathrm{and}\;j\;\mathrm{communicate},\\ 0, & \mathrm{otherwise}.\; \end{array}\right. \end{array}$$

### 6.4. Evaluation of Algorithm Robustness

We compare the sensitivity of our algorithm for different hyperparameters γ , maximum velocity $v_{max}$ , and maximum acceleration $a_{max}$ of the quadrotors in Table 6. It can be seen that the length of the trajectories does not vary much, and the minimum distance $d_{ij,mim}$ among quadrotors is always greater than the safe distance. Please note that as γ increases, $d_{ij,mim}$ gets closer to the safe distance, and the computation time gets shorter, which is consistent with the explanation in Section 3. In addition, the computation time of our algorithm is kept short. Therefore, our method has good robustness and can be applied to different scenarios.

## 7. Conclusions

In this paper, we propose a fully distributed algorithm for cooperative trajectory planning of quadrotor swarms based on DMPC-ADMM, which employs differential flatness property to handle the complex dynamics of the quadrotor. Additionally, we design a relaxed form of DCBF constraint to balance feasibility and safety. Due to the non-convexity of the DCBF, we linearize the DCBF at each time step and use an iterative convex optimization scheme to solve it. Simulation results show that our method can generate safe and smooth trajectories while satisfying dynamics constraints. Compared with the centralized strategy and several other distributed strategies in terms of computation time, safety, and feasibility, our method is more suitable for the trajectory planning of large-scale quadrotor swarms. Furthermore, the effect of the designed event-triggered mechanism for reducing the communication overhead is also verified.

In future work, we will improve the event-triggered mechanism to enhance inter-quadrotor communication efficiency. It is also worth exploring how to improve the robustness of the algorithm considering the presence of uncertainties in practice, such as perceptual uncertainty, communication packet loss, and external perturbations. Additionally, considering that the trajectory planning framework presented in this paper is currently implemented synchronously, limiting its flexibility, we will develop an asynchronous implementation.

## Figures and Tables

**Table 2:** Parameters setting.

Parameters	Values	Parameters	Values
*H*	15	*P*	$50\cdot I_{10}$
$T_{s}$	0.08 s	*Q*	$50\cdot I_{10}$
$\gamma_{io},\gamma_{ij}$	0.6	*R*	$1\cdot I_{4}$
*r*	0.2 m	*S*	$1\cdot I_{4}$
$\Delta^{\mathrm{dect}}$	20 m	$\epsilon_{abs},\epsilon_{1},\epsilon_{2}$	0.01
ρ	1	$d_{max}$	50
α	1 × 10^8^	$l_{max}$	20

**Table 3:** Comparison of the simulation results of the four methods for ten quadrotors cross a finite space with obstacles. The safe distance among quadrotors is $d_{ij,safe}=0.4\;m$ and the safe distance between quadrotors and obstacles is $d_{io,safe}=0.2\;m$ .

Coordination Strategy	Trajectory Length (m)				$d_{\mathrm{ij},\min}$ (m)	$d_{\mathrm{io},\min}$ (m)	Av. comp_time (ms)
	min	max	av.	std.			
CMPC	44.09	44.41	44.28	0.12	1.04	0.22	546.6
CVMPC	44.11	44.87	44.44	0.22	1.22	0.15	57.6
DMPC	44.18	44.67	44.42	0.19	1.12	0.17	47.1
MPC-ADMM (ours)	44.09	44.55	44.30	0.14	1.13	0.21	7.7

**Table 4:** Comparison of the simulation results of the four methods for eight quadrotors exchanging positions flight. The safe distance among quadrotors is $d_{ij,safe}=0.4\;m$ .

Coordination Strategy	Trajectory Length (m)				$d_{\mathrm{ij},\min}$ (m)	Av. comp_time (ms)
	min	max	av.	std.		
CMPC	12.14	12.41	12.22	0.09	0.45	293.7
CVMPC	12.17	12.68	12.45	0.20	0.15	73.7
DMPC	12.08	13.07	12.45	0.30	0.28	74.8
MPC-ADMM (ours)	12.06	12.62	12.30	0.21	0.48	16.1

**Table 5:** Comparison of the four methods in terms of average computation time (Av), collision probability (Cp), and feasibility (Fea) in Scenario 2.

Coordination Strategy	2 Quadrotors			4 Quadrotors			8 Quadrotors			16 Quadrotors		
	Av (ms)	Cp (%)	Fea	Av (ms)	Cp (%)	Fea	Av (ms)	Cp (%)	Fea	Av (ms)	Cp (%)	Fea
CMPC	12.2	0	Yes	61.9	0	Yes	293.7	0	Yes	1301.9	3	Yes
CVMPC	24.1	0	Yes	47.5	13	Yes	73.7	56	No	104.1	85	No
DMPC	23.6	0	Yes	40.2	0	Yes	74.8	21	Yes	101.3	59	No
MPC-ADMM (ours)	3.5	0	Yes	6.0	0	Yes	16.1	2	Yes	44.4	8	Yes

**Table 6:** Performance comparison of our algorithm for eight quadrotors exchanging positions flight at different hyperparameter $\gamma_{ij}$ , maximum velocity $v_{max}$ (m/s) and maximum acceleration $a_{max}\left(m/s^{2}\right)$ . The safe distance among quadrotors is $d_{ij,safe}=0.4\;m$ .

Parameters	Trajectory Length (m)				$d_{\mathrm{ij},\min}$ (m)	Av. comp_time (ms)
	min	max	av.	std.		
$\gamma_{ij}=0.4,v_{max}=3,a_{max}=1$	12.07	12.58	12.30	0.20	0.62	16.4
$\gamma_{ij}=0.6,v_{max}=3,a_{max}=1$	12.06	12.62	12.30	0.21	0.48	16.1
$\gamma_{ij}=0.8,v_{max}=3,a_{max}=1$	12.05	12.43	12.24	0.14	0.47	14.4
$\gamma_{ij}=1.0,v_{max}=3,a_{max}=1$	12.03	12.49	12.18	0.15	0.41	10.2
$\gamma_{ij}=0.6,v_{max}=5,a_{max}=1$	12.07	12.83	12.32	0.25	0.56	15.4
$\gamma_{ij}=0.6,v_{max}=7,a_{max}=1$	12.09	12.89	12.33	0.27	0.51	16.7
$\gamma_{ij}=0.6,v_{max}=5,a_{max}=2$	12.12	13.12	12.57	0.35	0.53	16.2
$\gamma_{ij}=0.6,v_{max}=5,a_{max}=4$	12.67	13.27	12.95	0.25	0.60	12.7

## Data Availability

Data are contained within the article.

## Abbreviations

The following abbreviations are used in this manuscript:

UAV	Unmanned Aerial Vehicle
MPC	Model Predictive Control
ADMM	Alternating Direction Multiplier Method
DCBF	Discrete-time CBF
MILP	Mixed Integer Linear Programming
MIQP	Mixed Integer Quadratic Programming
SCP	Sequential Convex Programming
ORCA	Optimal Reciprocal Collision Avoidance
AGV	Autonomous Guided Vessel
CMPC	Centralized MPC
CVMPC	Constant Velocity MPC
DMPC	Distributed MPC
**Symbols**	**Definitions**
z	Flat state of the quadrotor, including position, velocity, acceleration, and yaw angle.
ζ	Flat output of the quadrotor, including position and yaw angle.
v	Flat input of the quadrotor, including the third-order derivatives of the position and yaw rate.
$\Delta^{\mathrm{dect}}$	Maximum distance that quadrotors can communicate.
$N_{i}$	Set of neighbors of quadrotor *i*.
O	Set of obstacles.
Ω	Dynamics constraint of the quadrotor.
*H*	Prediction horizon of the optimization problem.
$w_{i}$	Duplicate of the trajectory $\zeta_{i}$ .
$w_{i\to j}$	Duplicate of quadrotor *i* for the trajectory $\zeta_{j}$ of quadrotor *j*.
Φ	All the constraints in (12) except for inter-quadrotor collision avoidance.
$DCBC_{ij}$	Safety constraints between quadrotor *i* and quadrotor *j*.
$DCBC_{io}$	Safety constraints between quadrotor *i* and obstacle *o*.
